# Small RNA Sequencing of Aqueous Humor and Plasma in Patients With Primary Open-Angle Glaucoma

**DOI:** 10.1167/iovs.62.7.24

**Published:** 2021-06-22

**Authors:** Wouter H. G. Hubens, Julian Krauskopf, Henny J. M. Beckers, Jos C. S. Kleinjans, Carroll A. B. Webers, Theo G. M. F. Gorgels

**Affiliations:** 1 University Eye Clinic Maastricht, Maastricht University Medical Center, Maastricht, The Netherlands; 2 School for Mental Health and Neuroscience, Maastricht University, Maastricht, The Netherlands; 3 Department of Toxicogenomics, Maastricht University, Maastricht, The Netherlands

**Keywords:** aqueous humor, microRNA, plasma, primary open-angle glaucoma, small RNA sequencing

## Abstract

**Purpose:**

Identify differentially expressed microRNAs (miRNAs) in aqueous humor (AH) and blood of primary open-angle glaucoma (POAG) patients by using small RNA sequencing. These may provide insight into POAG pathophysiology or serve as diagnostic biomarker.

**Methods:**

AH and plasma of nine POAG patients and 10 cataract control patients were small RNA sequenced on Illumina NovaSeq 6000. Identification of gene transcripts targeted by differentially expressed miRNAs was done with miRWalk and MirPath. These targets were used for pathway analysis and Gene Ontology enrichment. Diagnostic potential was evaluated by receiver operating characteristics analysis.

**Results:**

We identified 715 miRNAs in plasma and 62 miRNAs in AH. Plasma miRNA profile did not differ between POAG and control. In contrast, in AH, seven miRNAs were differentially expressed. Hsa-miR-30a-3p, hsa-miR-143-3p, hsa-miR-211-5p, and hsa-miR-221-3p were upregulated, whereas hsa-miR-92a-3p, hsa-miR-451a, and hsa-miR-486-5p were downregulated in POAG. Compared to previous studies, hsa-mir-143-3p, hsa-miR-211-5p, and hsa-miR-221-3p were reported previously, strengthening their involvement in POAG whereas hsa-miR-30a-3p, hsa-miR-92a-3p, and hsa-miR-486-5p are implicated in POAG for the first time. Identified gene transcripts were involved in several pathways, some implicated in glaucoma before (e.g., TGF-β and neurotrophin signaling), whereas others are new (e.g., prolactin and apelin signaling). In respect to diagnostics, AH concentration of hsa-mir-143-3p had an area under the curve (AUC) of 0.889. Combined with hsa-miR-221-3p, AUC improved to 0.96.

**Conclusions:**

Small RNA sequencing identified seven differentially expressed miRNAs in AH of POAG patients. The differentially expressed miRNAs may be useful as POAG biomarkers or could become targets for new therapeutic strategies.

Glaucoma is the leading cause of irreversible blindness, currently estimated to affect between 75 million and 80 million patients worldwide.^1^^,^^2^ The most common form is primary open-angle glaucoma (POAG).^3^ The exact pathology is unknown, yet several risk factors have been identified, including intraocular pressure (IOP), aging, and a positive family history.^2^^,^^4^^–^^7^ Lowering IOP of glaucoma patients is currently the only proven treatment strategy.^8^ Although this has an effect in most patients, still, at the end of life, approximately 24% of patients are unilaterally blind and 10% bilaterally blind.^9^ It is therefore important to learn more about the pathophysiology, because this may lead to new treatment options.

MicroRNAs (miRNAs), short noncoding RNA fragments of approximately 22 nucleotides, have been investigated as potential contributors to POAG pathology, because they can regulate many cellular processes, via transcriptional inhibition or targeted messenger RNA (mRNA) degradation.^10^^–^^13^ Microarrays and sequencing studies identified several miRNAs with an altered expression in aqueous humor (AH) of glaucoma patients compared to controls.^14^^–^^18^ One of these studies performed small RNA next-generation sequencing to enable a comprehensive analysis of miRNA changes in POAG.^14^

Differentially expressed miRNA may also serve as a diagnostic biomarker for glaucoma. Early diagnosis of glaucoma is of vital importance to preserve vision. In this respect, it is also of interest to identify differentially expressed miRNAs in blood, which is more easily obtained than AH. AH is only collected during intraocular surgery. For instance, Liu et al.^19^ performed small RNA sequencing in serum of glaucoma patients and identified hsa-miR-210-3p as a possible biomarker with an area under the curve (AUC) of 0.86 in a receiver operating characteristics (ROC) analysis.

In the current study, we performed small RNA sequencing in AH and plasma of POAG patients and, as controls, nonglaucomatous cataract patients to identify differentially expressed miRNAs. In addition, because plasma and AH were obtained from the same patients, we could compare the results on the two body fluids.

## Methods

### Samples

Blood plasma samples and AH were obtained from the Eye Tissue Bank Maastricht (ETBM). The ETBM collects and stores biomaterial from glaucoma and cataract patients visiting the University Eye Clinic Maastricht. AH was collected at the start of cataract extraction surgery or at the start of combined cataract-minimally invasive glaucoma surgery and immediately snap-frozen in liquid nitrogen. The ETBM and the current study adhered to the tenets of the Declaration of Helsinki. All participating patients had signed informed consent for the collection and use of their biomaterial for scientific studies. The medical-ethical committee of Maastricht University Medical Center approved the current study (approval number 2018-0647).

### Criteria

We obtained AH and plasma from nine POAG patients and from 10 cataract patients who served as our control group. Glaucoma specialists diagnosed POAG using the following criteria: open-anterior chamber angles on gonioscopy; no physical abnormalities in the anterior chamber (pigmentation or pseudoexfoliation); glaucomatous optic nerve head. Untreated IOP of included POAG patients was higher than 21 mm Hg to exclude normal tension glaucoma. All samples were from patients who had no severe coexisting systemic disease(s) (i.e., diabetes, cancer, chronic obstructive pulmonary disease, or severe obesity, i.e., body mass index [BMI] >35) and had no history of ocular diseases (other than glaucoma and cataract). AH was collected at the start of cataract surgery. In POAG patients surgery was combined with placement of trabecular micro-bypass stent(s) (iStent; Glaukos Corp, San Clemente, CA, USA) to further reduce IOP. AH and plasma were collected on the same day.

### Small RNA Sequencing

AH and plasma miRNAs were isolated using the miRNeasy Serum/Plasma Kit (Qiagen, Hilden, Germany). RNA quality and yield were assessed by means of the Bioanalyzer 2100 using the small RNA Kit (Agilent Technology, Tokyo, Japan). Libraries were prepared for sequencing using the Perkin Elmer small RNA-seq v3 with UDI kit (Perkin Elmer, New York, NY, USA) and subsequently sequenced using the Illumina NovaSeq 6000 sequencing platform (Illumina, San Diego, CA, USA).

After quality control using FastQC (version 0.11.9), data were processed using miRGe2^20^ with the latest version of miRBase^21^ as described earlier.^22^ The resulting expression matrix was analyzed using open-source software R version 4.0.^23^ Data normalization and screening for differential expression was done separately for AH and plasma using DESeq2 package.^21^ Briefly, miRNAs were included when measured in minimally 60% of subjects. Missing values were imputed by k-nearest neighbor imputation. On the remaining, normalized miRNAs, a negative binominal test (glaucoma vs. control) was performed, thereby correcting for gender and day of library preparation (batch effect) in AH. For plasma, we additionally corrected for age, smoking, and BMI. These confounders were identified by running a negative binominal test to evaluate the impact of a confounder on the expression matrix. MiRNAs were considered significant between the two groups when showing an adjusted *P* value < 0.2 (adjusted for multiple testing using Benjamini-Hochberg method as implemented in DESeq2).

### Target Genes and Pathway Analysis

We used two approaches to find genes whose transcripts are potential targets of the differentially expressed miRNAs. In the first approach, transcripts were identified using the miRWalk 3.0 database (http://zmf.umm.uni-heidelberg.de/apps/zmf/mirwalk2),^24^ as described previously.^25^ We filtered for gene transcripts with a miRTarBase entry, as for those genes an interaction with the miRNA is experimentally validated by reporter assay, Western blot, or in microarray studies.^26^ Next, these validated genes were analyzed using the Enrichr software (http://amp.pharm.mssm.edu/Enrichr/),^27^ an integrative web-based software application, for their overrepresentation in biological pathways. In our study, KEGG (Kyoto Encyclopedia of Genes and Genomes, http://www.genome.jp/kegg/) pathways were investigated and Gene Ontology (GO) biological processes. Pathways and GO with a *P* value < 0.05 (adjusted for multiple testing) were considered as significantly overrepresented.

For the second approach, differentially expressed miRNAs were uploaded to mirPath (version 3; http://snf-515788.vm.okeanos.grnet.gr), also for KEGG and GO analysis.^28^ Standard settings were used. In short, significance threshold is set to *P* < 0.05, including a FDR correction according to Fisher's exact test enrichment analysis. Heat maps and tables were downloaded of the generated pathways union (KEGG analysis) and generated categories union (GO analysis of biological processes)

### Biomarker Assessment

Differentially expressed miRNAs were assessed for their ability to distinguish glaucoma patients from nonglaucomatous controls by ROC analysis. ROC analysis was performed in GraphPad Prism 6 (GraphPad Software, San Diego, CA, USA) using the normalized miRNA expression.

## Results

### Clinical Information of the Patients

Clinical information of the nine POAG and ten cataract patients is listed in Table 1. Age was not significantly different between POAG and cataract control patients (*P* = 0.91). Prevalence of hypertension and hypercholesterolemia also did not differ between groups (*P* = 0.88 and 0.91). Average BMI of POAG patients (26.32 ± 2.26) was significantly higher than control patients (23.34 ± 3.08) (*P* = 0.04). Most POAG patients were on at least two types of topical IOP lowering medication and the treated IOP did not significantly differ from the IOP of control patients (*P* = 0.35). In respect to disease severity, our POAG group was heterogeneous, including patients with early, moderate, advanced and severe glaucoma,^29^ with on average a mean deviation on perimetry of −13.02 dB.

**Table 1.** Baseline Characteristics of Patients

Patient	Disease	Age (y)	Sex	BMI	mD (dB)	ROP (db/y)	IOP (mm Hg)	HT	HC	Topical Medication	Systemic Medication
466	POAG	84	M	25.64	−9.19	−0.45	10			Bimatoprost^*^; dorzolamide,^†^ timolol^‡^	Prednisolone,^¶^ alendronic acid
700	POAG	73	F	22.1	−1.79	0.61	18			Brinzolamide,^†^ timolol,^‡^ tafluprost^*^	None
576	POAG	80	M	28.41	−16.1	0.10	17	+	+	Dorzolamide,^†^ tafluprost^*^	Valsartan^**^
665	POAG	73	F	27.97	−2.68	−1.42	12	+	+	Latanoprost,^*^ brinzolamide,^†^ timolol,^‡^ polyvidon^§^	Irbesartan,^**^ metoprolol,^‡^ Rosuvastatin^††^
769	POAG	80	M	27.99	−26.43	0.20	20			Latanoprost,^*^ brinzolamide,^†^ brimonidine^ǁ^	None
810	POAG	69	F	26.99	−11.1	−0.31	18	+		None (intolerant)	Metoprolol,^‡^ valsartan,^**^ simvastatin^††^
464	POAG	70	F	27.44	−5.57	−1.36	21			Timolol,^‡^ travoprost^*^	None
615	POAG	71	F	27.73	−25.31	0.55	10			Dorzolamide,^†^ timolol,^‡^ Bimatoprost^*^	None
1016	POAG	78	M	22.58	−18.98	−3.15	11			Dorzolamide,^†^ Timolol,^‡^ travoprost^*^	Atorvastatin,^††^ valsartan,^**^ lithium carbonate, mirtazapine,^‡‡^ quetiapine^§§^
412	Cataract	87	M	23.95	N/A	N/A	13	+		None	Losartan,^**^ amlodipine,^ǁǁ^ simvastatin,^††^ prednisolone^¶^
449	Cataract	86	M	17.99	N/A	N/A	16			None	Metoprolol,^‡^ valsartan,^**^ gemfibrozil^¶¶^
450	Cataract	81	V	21.08	N/A	N/A	22			None	Clopidogrel^##^
579	Cataract	83	M	23.5	N/A	N/A	16	+		None	Carbasalate calcium,^***^ metoprolol,^‡^ simvastatin,^††^ nifedipine^ǁǁ^
585	Cataract	84	M	20.41	N/A	N/A	16	+	+	None	Barnidipine,^ǁǁ^ enalapril,^†††^ metoprolol,^‡^ spironolactone^‡‡‡^
809	Cataract	60	M	24.49	N/A	N/A	18			None	None
447	Cataract	68	F	24.09	N/A	N/A	14		+	None	Simvastatin^††^
770	Cataract	75	F	24.09	N/A	N/A	20			None	Carbasalate calcium,^***^ irbesartan,^**^ verapamil,^ǁǁ^ ibandronic acid
1042	Cataract	79	M	23.39	N/A	N/A	10			None	None
1060	Cataract	55	M	30.39	N/A	N/A	18			None	None

Glaucoma disease severity was assessed with Humphrey visual field test, and expressed as the amount of mD dB. ROP is expressed as the dB loss per year. A positive rate of progression indicates that the patient scored better during the current visual field test in comparison to results of a previous test.

dB, decibels; HT, hypertension; HC, hypercholesterolemia; mD, mean deviation; ROP, rate of glaucoma disease progression.

* Prostaglandin analogue.

† Carbonic anhydrase inhibitor.

‡ Beta blocker.

§ Artificial tears.

ǁ α_2_-Agonist.

¶ Corticosteroid.

# Nonsteroidal anti-inflammatory drug.

** Angiotensin receptor blocker.

†† Statin.

‡‡ Nonselective serotonin reuptake inhibitor.

§§ Antipsychotic.

ǁǁ Calcium antagonist.

¶¶ Fibrate.

## P2Y12-inhibitor.

*** Antithromboticum.

††† Angiotensin converting enzyme inhibitor.

‡‡‡ Diuretic.

### Small RNA Sequencing Characteristics

**Figure 1.**
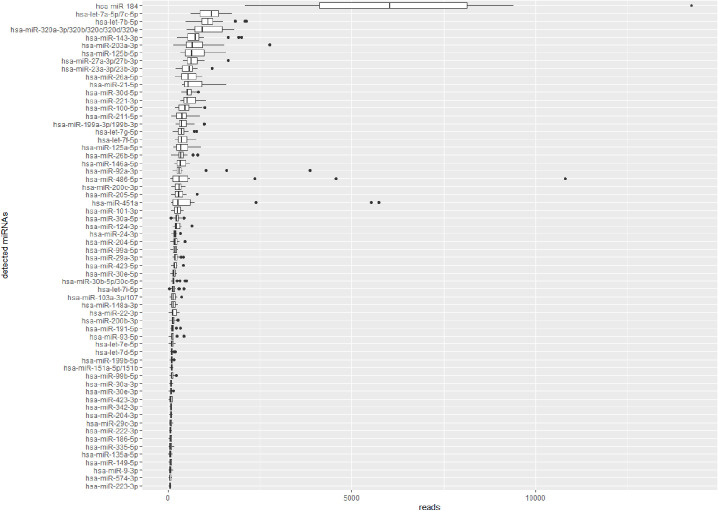
Next-generation sequencing of AH identified 62 miRNAs in AH of glaucoma patients and cataract patients. MiRNA expression is presented as median reads with interquartile range. *Dots* indicate values 1.5 times higher than the interquartile range.

Principle component analysis indicated that one AH sample (Supplemental Fig. S1a) and one plasma sample (Supplemental Fig. S1b), were strong outliers. We carefully checked the medical history for biological differences for these patients and the experimental details of these samples. Remarkably, these two samples deviated from the other samples in the amount of processed reads. The AH sample had much more processed reads than the other AH samples (1.2 million vs. on average 403,188 ± 224,757). On the other hand, the plasma sample had a distinctively lower amount of processed reads compared to the other plasma samples (4.1 million vs. on average 11.2 million ± 3.8 million). On the basis of these deviations, we therefore excluded these two samples from further analysis. For the remaining samples, reads were mapped to miRBASE entries. In AH, 262 miRNAs were identified (Supplemental File 1a) of which 62 were detected in at least 60% of the samples (Fig. 1; Supplemental File 1b). In plasma, 715 miRNAs were identified in at least 60% of the samples (Supplemental File 1c).

### Differential Expression and Pathway Analysis

**Figure 2.**
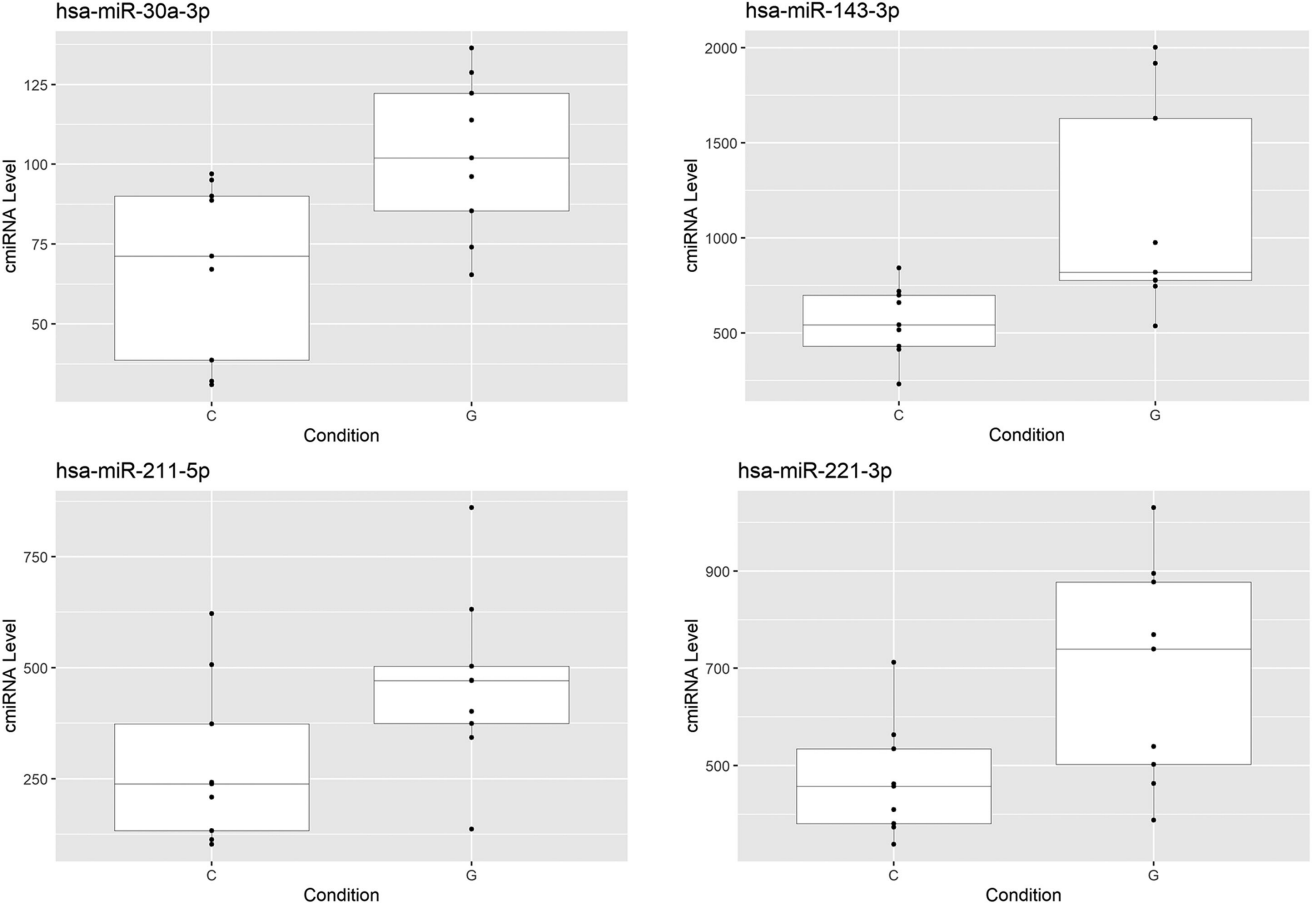
Upregulated miRNA in POAG (*G*) patients compared to controls (*C*).

**Figure 3.**
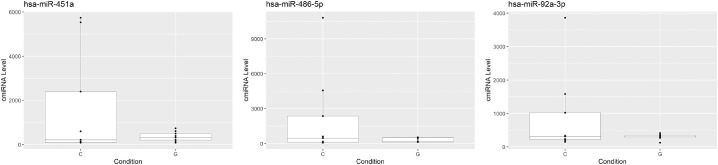
Downregulated miRNA in the aqueous humor of POAG patients (*G*) versus control patients (*C*).

Although we identified 715 miRNAs in plasma, none of these were significantly differentially expressed between POAG and control. On the other hand, of the 62 miRNAs detected in AH, seven miRNAs were significantly differentially expressed (*P* < 0.05, FDR corrected *P* < 0.2). Four were upregulated (Fig. 2) and three were downregulated in POAG (Fig. 3). As described in the Methods, we used two approaches to determine which gene transcripts are targeted by these miRNAs. The first method, by miRWalk, suggested 7129 genes with potential binding sites in their mRNA for these seven miRNAs (Supplemental File 2). For 410 gene transcripts, previous studies reported in miRTarBase, have experimentally validated binding of the miRNA. Based on these 410 validated targeted genes, gene enrichment analysis with Enrichr provided 60 significantly enriched KEGG (adjusted *P* < 0.05; Supplemental File 4a). Among these were several pathways previously implicated in glaucoma, such as neurotrophin signaling, fluid sheer stress, TNF signaling, and apoptosis. In addition, this analysis showed several strongly significant pathways (*P* < 0.01), which have not yet been connected with POAG, such as the prolactin signaling pathway and the apelin signaling pathway. The same gene set also provided 50 significantly enriched GO biological processes (adjusted *P* < 0.05; Supplemental File 4b). In line with the pathway analysis, cellular response to fluid sheer stress; cytokine mediation signaling pathway; generation of neurons; and regulation of apoptotic processes, were significantly enriched. GO enrichment also suggested an overrepresentation of biological processes related to transforming growth factor beta (e.g., regulation of transforming growth factor beta receptor signaling pathway).

Our second approach, using DIANA MirPath, suggested that the seven differentially expressed miRNAs interacted with mRNA transcripts of 2919 genes (Supplemental File 3). These genes significantly enriched 25 pathways (Supplemental Figure S2; Supplemental File 4c) and 94 biological gene ontology processes (Supplemental Fig. S3; Supplemental File 4d). Results were similar to our first approach and included pathways and GO processes related to apoptosis (e.g., p53 signaling), IOP regulation (e.g., TGF-β signaling, extracellular matrix remodeling) and immune response (e.g., cytokine-mediated signaling).

### Comparison of miRNA Composition of AH and Plasma

AH and plasma were obtained on the same day, which allowed for a fair comparison of miRNA expression in these two body fluids. MiRNA compositions clearly differed. Specifically, hsa-miR-184, the most abundant miRNA in AH (Fig. 1), was not detected in plasma, nor did we detect hsa-mir-124-3p in plasma. Conversely, several of the highly abundant miRNAs in plasma (top 40) were not detectable in any of the AH samples, such as hsa-miR-126-3p and hsa-miR-16-5p. Other abundant plasma miRNAs (top 20), while present in AH, had a much lower abundance in AH. For instance, hsa-miR-103a-3p/107 was the seventh most abundant plasma miRNA, whereas in AH it was ranked 40^th^.

### Correlation of AH miRNAs With Disease Characteristics

We studied whether the seven differentially expressed miRNAs were correlated with glaucoma disease characteristics. We tested IOP, disease severity (mean deviation) and rate of disease progression (mean deviation loss per year). Hsa-mir-143-3p correlated weakly with IOP (*R*^2^ = 0.251; *P* < 0.05). None of the expression levels correlated significantly with disease severity or disease progression.

### AH miRNAs as Biomarker

**Figure 4.**
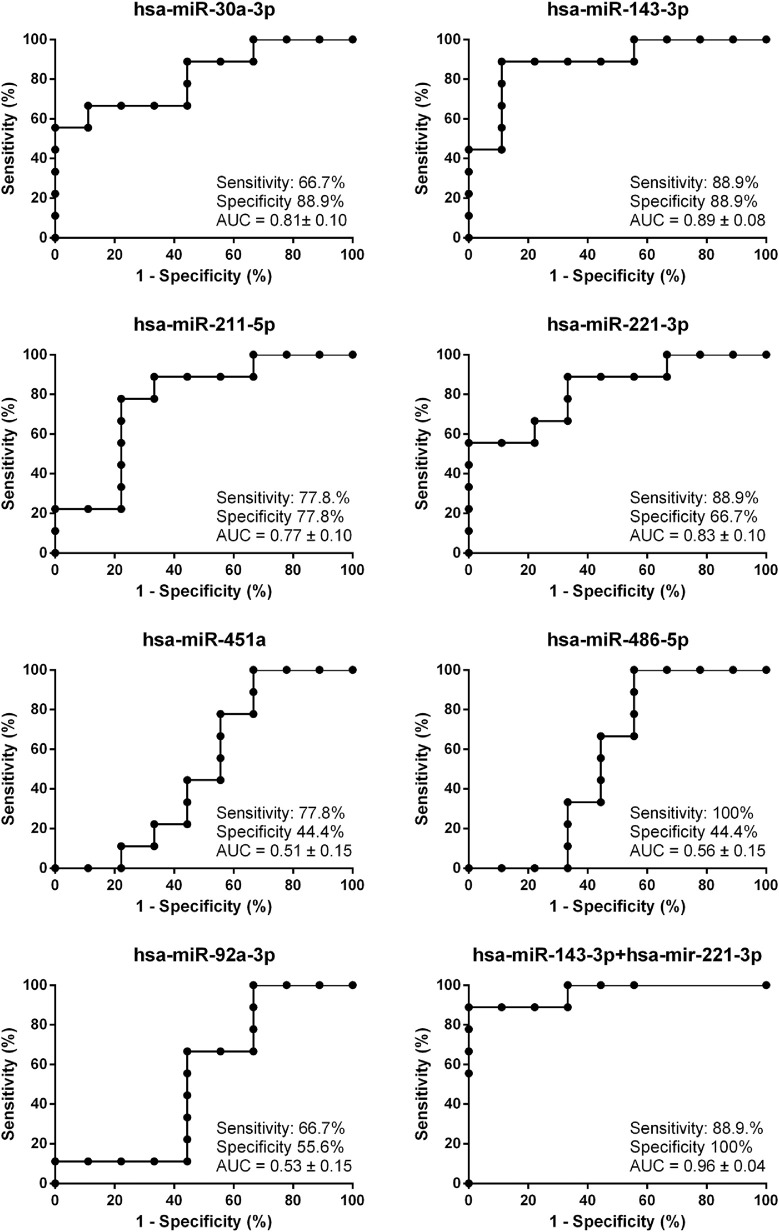
AUC for ROC analysis of each of the seven differentially expressed miRNAs. Of the possible combination of miRNAs that were assessed, the combination of hsa-miR-221-3p with hsa-miR143-3p had the best glaucoma detection.

To evaluate whether differentially expressed AH miRNAs are potential diagnostic biomarkers, ROC curves were created for each of the seven differentially expressed miRNAs (Fig. 4). The upregulated miRNAs had AUC values between 0.77 and 0.89, suggesting they might have potential as diagnostic biomarkers. In contrast, for the downregulated miRNAs, it was not possible to distinguish glaucoma patients from controls based on their expression levels, showing AUC values close to 0.5.

## Discussion

In the current study, we performed small RNA sequencing on AH and plasma of POAG patients and controls to identify differentially expressed miRNAs that could provide more insight into glaucoma pathophysiology and potentially serve as disease biomarkers. Although we identified 715 miRNAs in plasma, none were differentially expressed. In contrast, of 62 miRNAs identified in AH, seven were significantly differentially expressed between POAG patients and controls.

### Previous microRNA Studies

To our knowledge there are five previous studies that investigated the AH miRNA profile of glaucoma patients comprehensively, using untargeted techniques such as microarrays and sequencing (Table 2).^14^^–^^18^ These studies reported in total 129 regulated microRNAs. Remarkably, only for one microRNA (hsa-miR-4725-3p), the differential expression could be replicated in a second study. The differential expression of all other microRNAs have not been replicated yet. Several reasons could be postulated for the lack of agreement between studies. Technical differences between sequencing and microarray hybridization can influence the results, as reviewed by Git et al.^30^ Results are also influenced by sample preparation differences. For instance, it is important to mention whether samples are spun in a centrifuge before miRNA isolation, because AH may contain cell-free circulating miRNAs that originate from debris or dying cells,^31^^,^^32^ as well as miRNAs contained in vesicles such as exosomes.^33^ Obviously, differences in patient selection such as presence of systemic diseases or medication, as well as differences in the type of glaucoma and glaucoma parameters, that is, age, rate of disease progression, and disease severity, also contribute to discrepancies. In view of the low level of agreement between previous studies, we focused on controlling variation in our experiment. With respect to patient selection, we applied very strict criteria resulting in well-defined homogeneous groups. In addition, because AH samples were collected during cataract surgery (control patients) or during a cataract surgery combined with placement of trabecular micro-bypass stents (POAG patients), the conditions of AH collection were highly comparable between groups. AH samples were of high quality because they were immediately snap-frozen in liquid nitrogen in the operating theater. By analyzing these samples with small RNA-sequencing technology, we obtained an unbiased comprehensive overview of AH miRNAs. We found seven differentially expressed AH miRNAs. Importantly, we were able to replicate the significant upregulation of hsa-miR-143-3p^16^ and hsa-miR-221-3p.^14^ Apart from the five untargeted studies mentioned above, there are also targeted studies on AH miRNA, using e.g. RT-PCR. We checked whether our differentially expressed miRNAs were measured in any of these studies. We found that upregulation of hsa-miR-211 has been reported previously.^34^ Thus our study strengthened the involvement of three specific miRNAs in POAG pathophysiology. On the other hand, we found conflicting results for hsa-miR-451a. In our study, hsa-miR-451a was significantly downregulated, whereas Drewry et al.^17^ reported a significant upregulation. As discussed above, differences in the technology used or patient selection criteria (e.g., Drewry did not provide information regarding systemic diseases, and six of 12 POAG patients were classified as having normal-tension glaucoma based on untreated IOP <21 mm Hg) may contribute to this discrepancy.

**Table 2.** Comparison of the Seven Differentially Expressed miRNAs In Our Study and Previously Published miRNA Studies on Glaucoma

Technique	Our Study Small RNA-Seq	Liu et al.^14^ Small RNA-Seq	Tanaka et al.^15^ 3D-Gene Human miRNA ver.1.60 chip (2019 miRNAs)	Jayaram et al.^16^ 2 TaqMan Low-Density Human MicroRNA Arrays(754 miRNAs)	Drewry et al.^17^ NanoString nCounterHuman v3 miRNA Expression Assay(800 miRNAs)	Hindle et al.^18^ Custom miR-Finder Array(372 miRNAs)
Sample size (C vs. G)	9 vs. 9	6 vs. 6	10 vs. 10	8 vs. 6	11 vs. 12	31 vs. 17
Glaucoma type	POAG no comorbidity	POAG no comorbidity	POAG, PACG, PEX	POAG with comorbidities	POAG (perhaps NTG)	POAG, PEX
Detected miRNAs	262	>426	530.5 ± 44.6	338	298	239
Significant miRNAs	7 (of 62)	88	29	3	3	6 (of 20)
hsa-mir-30a-3p	↑	Unknown	NS	NS	N/A	NS
hsa-mir-143-3p	↑	Unknown	N/A	↑	N/A	NS
hsa-miR-221-3p	↑	↑	N/A	N/A	N/A	N/A
hsa-mir-211-5p	↑	Unknown	NS	NS	NS	N/A
hsa-mir-486-5p	↓	NS	N/A	N/A	N/A	N/A
hsa-mir-451a	↓	Unknown	NS	NS	↑	NS
hsa-mir-92a-3p	↓	Unknown	NS	NS	N/A	N/A

Studies are indicated by name of the first author. Unknown indicates that we are uncertain whether the miRNA was detected in the study (if it was, it was not significant), because the authors did not disclose the full list of miRNAs identified.

C, control; G, glaucoma; ↑, significantly upregulated; ↓, significantly downregulated; N/A, not available for detection in the array; NS, detected in study but not significant.

With respect to blood, only one other study analyzed blood of glaucoma patients by small RNA sequencing. Liu et al.^19^ reported three significantly upregulated miRNAs in serum: hsa-miR-210-3p, hsa-miR-885-5p and hsa-miR-3149, of which hsa-miR-210-3p was validated with qPCR in a larger cohort of 59 POAG and 59 controls. In contrast to their results, we did not find any significantly differentially expressed miRNAs in plasma. Hsa-miR-210-3p and hsa-mir-885-5p were slightly lower in our glaucoma patients compared to controls (fold changes of 0.87 and 0.86, respectively), and we did not detect hsa-miR-3149. A drawback of small RNA sequencing may be that protocols for data analysis are not yet standardized. This might contribute to discrepancies observed between the two studies. For instance, we used miRGe2 for analysis and corrected for potential confounders, whereas the study of Liu et al.^19^ used NOISeq and did not mention any corrections.

### Biological Function of the Differentially Expressed miRNAs

It is uncertain whether AH miRNAs actively contribute to POAG c.q. exert a biological function, for example after entering cells of the trabecular meshwork (TM). There is proof-of-principle for this, for example, topical administration of miR-21-5p mimics increased miR-21-5p expression in TM and reduced IOP by 17%.^35^ Yet, it remains to be investigated whether the copy number of naturally present miRNAs is sufficient for a significant influence. If we assume that they are functional and do regulate ocular gene expression, then it is important to unravel which biological processes are affected to better understand POAG pathology and find new therapeutic strategies.

We assessed the functionality of the differentially expressed miRNAs in several ways. We first checked whether they correlated with disease characteristics, that is, IOP, disease severity (mean deviation), and rate of disease progression (mean deviation loss per year). Interestingly, we found that upregulation of hsa-mir-143-3p correlated weakly yet significantly, with IOP (*R*^2^ = 0.251; *P* < 0.05). In mice, expression of mmu-miR-143 was 100-1000 times higher in TM compared to other ocular tissues.^36^ Targeted deletion of the mir-143/miR-145 cluster resulted in an approximately twofold increased outflow capacity and reduction of IOP. Hypothetically, increased levels of this miRNA observed in POAG AH may target TM cells and contribute to the decreased outflow capacity, c.q. increased IOP in glaucoma patients.

Second, we assessed the biological function of our differentially expressed miRNAs using bioinformatics approaches. Bioinformatics can predict which gene transcripts and which molecular pathways are targeted. Depending on the database used, the list of targeted gene transcripts varied greatly. We used two approaches (miRWalk and miRPath), which reported 410 and 2919 targets, respectively. Only 203 genes overlapped between these two analysis methods. Despite these differences, both analysis methods agreed on enrichment of several pathways known to be related to glaucoma, for example, apoptosis, inflammation, TGF-beta signaling and fluids sheer stress. Pathway analysis of target genes identified with miRWalk, also pointed to enrichment of neurotrophin signaling pathways, in line with previous studies.^17^^,^^18^ In addition, this analysis suggested a potential involvement of the prolactin pathway and the apelin pathway, which have not been implicated in POAG pathology before. Recently, it has been discovered that prolactin protects the retinal pigment epithelium and photoreceptors from age-related deficiencies and age-related oxidative stress.^37^^,^^38^ Interestingly, prolactin and the prolactin receptor are also expressed in retinal ganglion cells (RGC).^39^ Because POAG is an age-related disease, perhaps it will be of interest to investigate the role of prolactin in RGC degeneration. With respect to the apelin signaling pathway, studies have reported a decrease in apelin in serum of exfoliation glaucoma patients.^40^ In addition, in mice exposed to glutamate excitotoxicity via N-methyl-D-aspartate, apelin is neuroprotective, preventing retinal ganglion cell death.^41^^–^^43^

In addition, we investigated whether experimental data are available in the literature regarding the ocular function and potential role in glaucoma pathophysiology (summarized in Table 3). As mentioned previously, hsa-mir-143-3p, as part of the miR-143/miR-145 cluster, is important for regulation of the outflow capacity of the TM.^36^ Interestingly, hsa-miR-143-3p is located on the long arm of chromosome 5 (5q32), a locus associated with increased risk for development of POAG.^44^

**Table 3.** Overview of Our Differentially Expressed miRNAs in POAG AH

miRNA	POAG Expression	Validation	Ocular Function and Possible Relation to POAG Pathology
hsa-mir-143-3p	Upregulated	In agreement with microarray^14^	Important for regulating the outflow capacity of the TM.^36^ Located on the long arm of chromosome 5 (5q32), a locus associated with POAG.^44^ In our study, we found a weak correlation between IOP and hsa-miR-143-3p concentration (*R*^2^ = 0.251; *P* < 0.05)
hsa-mir-211-5p	Upregulated	In agreement with RT-PCR^34^	Retinal levels correlate with IOP, mediated by oxidative stress and induced RGC apoptosis.^34^ In lens it can target Sirt1.^47^ Decreased expression of SIRT1 has been observed in the TM of glaucoma patients.^48^
hsa-miR-221-3p	Upregulated	In agreement with Small RNA sequencing^14^	Pro-apoptotic in lens epithelial cells of cataract patients.^49^^,^^50^ Pro-apoptotic effects in RGCs of POAG patients requires further investigation.
hsa-mir-30a-3p	Upregulated	Novel finding	May inhibit the production of brain derived neurotrophic factors, perhaps contributing to neurotrophin deprivation observed in POAG.^51^^–^^53^
hsa-mir-451a	Downregulated	Conflicting with a microarray reporting upregulation^16^	Enhances mitochondrial function in the retina by downregulating ATF2.^54^ Decreases expression of MMP2,^54^ a protein with observed increased expression in POAG patients.^58^
hsa-mir-486-5p	Downregulated	Novel finding	Targets Smad2, which inhibits ECM remodeling in the lens.^59^ In TM, Smad2 prevents mechanical stress induced autophagy. Effect of this miRNA on ECM remodeling in POAG TM,^60^ and on autophagy of TM cells requires further investigation.
hsa-mir-92a-3p	Downregulated	Novel finding	May inhibit inflammation and is downregulated during inflammatory responses.^62^^,^^63^ May play a role in retinal cell survival.^64^

We searched the literature for studies that previously reported these microRNAs regulated in POAG, validating the results. If miRNA was not a novel finding, we indicated the method that was used previously. In addition, we speculated on their ocular function and potential involvement in POAG.

With respect to hsa-miR-211-5p, Yang et al.^34^ reported that expression of miR-211 was increased in RGC cells exposed to oxidative stress. As reviewed elsewhere, oxidative stress plays an important role in the pathophysiology of glaucoma.^45^^,^^46^ Elevated levels of miR-211, caused downregulation of fibroblast growth factor receptor substrate 2 signaling and triggered cell death in RGCs.^34^ A similar proapoptotic effect was observed in the lens, by targeting NAD+-dependent histone deacetylase sirtulin 1 (SIRT1).^47^ Interestingly, a decreased expression of SIRT1 has been observed in the TM of glaucoma patients.^48^

Upregulation of hsa-miR-221-3p is also pro-apoptotic in ocular tissue, as shown by two studies with lens epithelial cells of cataract patients.^49^^,^^50^ Further studies are required to assess whether hsa-miR-211-5p and hsa-miR-221-3p circulating in AH can indeed exert a proapoptotic effect on TM cells or if they reflect the increased apoptosis of RGCs.

Our study implicates hsa-miR-30a-3p with glaucoma for the first time. The hsa-miR-30 family can inhibit the production of brain-derived neurotrophic factors.^51^ This suggests that the observed upregulation of hsa-miR-30a-3p may contribute to neurotrophin deprivation in glaucoma patients.^52^^,^^53^

A recent study explored the role of miR-451a in diabetic retinopathy.^54^ This study reported a decrease of mmu-miR-451a in the retina of Akita mice, a type 1 diabetic animal model, as well as in primary human retinal pigment epithelial (RPE) cells and ARPE-19 cells exposed to diabetic conditions. They subsequently showed that miR-451a regulates mitochondrial function of RPE cells. Transfecting ARPE-19 cells with miR-451a inhibitors decreased mitochondrial function whereas miR-451a mimics improved mitochondrial function. On the basis of these data, it would be of interest to investigate further whether the decreased expression of hsa-miR-451a in AH of glaucoma patients could contribute to mitochondrial dysfunction. Mitochondrial dysfunction has been suggested to play a role in glaucoma pathophysiology.^55^^,^^56^ Last, Shao et al.^54^ investigated potential targets of miR-451a. They identified *ATF2,* a transcription factor that regulates the expression of several proteins, as a target of miR-451a. One of the proteins with altered expression by miR-451a through ATF2 was MMP2, i.e. miR-451a inhibition resulting in increased expression of MMP2. Our observation of decreased expression of hsa-miR-451a in AH of glaucoma patients is in line with previous reports of increased expression of MMP2 in AH.^57^^,^^58^

Hsa-miR-486-5p was downregulated in glaucoma patients in our study. In the lens, hsa-miR-486-5p is shown to suppress TGF-β2 induced extracellular matrix (ECM) remodeling by targeting Smad2.^59^ If this miRNA also targets SMAD2 in TM cells, this may contribute to the increased ECM remodeling observed in glaucoma patients e.g. increase production of fibronectin that leads to increased stiffness and reduced AH outflow.^60^ In addition to ECM remodeling, SMAD2 also plays an important role in autophagy of TM cells in response to mechanical stress.^61^ Downregulation of SMAD2 prevented mechanical stress induced autophagy. A future study could investigate if restoring hsa-miR-486-5p to normal levels, could decrease SMAD2 signaling in TM cells, preventing ECM remodeling and prevent mechanical stress induced cell death.

Little is known regarding the ocular function of hsa-miR-92a-3p. One study reported a significant downregulation in the corneal epithelium after exposure to bacterial antigens.^62^ This suggests that miR-92 plays a role in inflammation. Indeed, it can inhibit inflammatory response by targeting MKK4 kinase, and its expression is reduced in macrophages on activation of toll-like receptors.^63^ On the other hand, a study with rod photoreceptors reported that a cluster of miRNAs, including miR-92a, was downregulated in photoreceptors of mice with conditional knock-out of *Dicer1*, suggesting that it plays an important role in cell survival.^64^ More studies are needed to elucidate whether a decrease in hsa-miR-92a-3p in AH is related to the increased inflammation in glaucoma patients,^65^ or if it reflects the decreased survival of RGCs.

It is relatively easy to synthesize antagomirs for upregulated miRNAs or agomirs for downregulated microRNAs. In fact, as described above, for some of the differentially expressed miRNAs, these are already available and tested. The use of these (ant)agomirs, both in vivo and in vitro, is a promising approach to elucidate the ocular function of these seven differentially expressed miRNAs further, which may lead to the discovering novel candidate drugs to treat glaucoma. The therapeutic potential of miRNAs is already highlighted by the example of topical administration of miR-21-5p, which resulted in IOP reduction.^35^

### Differentially Expressed miRNAs as Diagnostic Biomarkers

A second aim of our study was to determine whether differentially expressed miRNAs may also serve as a diagnostic biomarker for glaucoma. Although we, unfortunately, did not find differentially expressed miRNAs in the plasma, our analysis of AH revealed seven differentially regulated miRNAs. Several of these had a decent sensitivity and specificity to distinguish glaucoma patients from controls, suggestive for diagnostic potential (Fig. 4). Expression levels of the novel reported hsa-miR-30a-3p had a ROC of 0.81. The other three miRNAs with significant upregulation had similar ROC values, ranging from 0.77 (hsa-miR-211-5p) to 0.89 (hsa-mir-143-3p). In contrast, expression levels of the three novel reported downregulated miRNAs had no ability to distinguish glaucoma patients from controls, with ROC values ranging from 0.51 (hsa-miR-451a) to 0.56 (hsa-miR-486-5p). We additionally investigated if a combination of miRNAs would improve their utility as biomarkers. Combination of hsa-miR-221-3p and hsa-miR-143-3p had the highest diagnostic potential, with a ROC of 0.96. Although these results are promising, our sample size was limited, and future studies with a larger number of patients are needed to confirm the value of this miRNA combination as a biomarker for POAG diagnosis. In addition, as collecting AH is an invasive procedure, normally only performed during planned surgeries, the utility of these two miRNAs for routine diagnostics is currently limited. With the improvements in techniques to analyze AH noninvasively, that is, without taking a biopsy, this might change in the future. For instance, Raman spectroscopy, although still requiring optimization, holds promise to detect aqueous humor content.^66^^–^^68^ With specific protocols this technique was also able to detect miRNAs in vitro.^69^

In conclusion, small RNA sequencing revealed seven miRNAs with differential expression in AH of POAG patients compared to cataract controls, while no differences were observed in plasma. For three miRNAs, their differential expression in AH is here implicated in POAG pathology for the first time. The differentially expressed miRNAs may prove useful as POAG biomarkers or could provide targets for new therapeutic strategies, meriting further investigation in larger studies.

## Supplementary Material

Supplement 1

Supplement 2

Supplement 3

Supplement 4

Supplement 5
