# Supplementary material for: Small RNA Sequencing of Aqueous Humor and Plasma in Patients With Primary Open-Angle Glaucoma
Source: Invest Ophthalmol Vis Sci. 2021 Jun 22;62(7):24. doi: 10.1167/iovs.62.7.24 (PMC8237107; doi:10.1167/iovs.62.7.24)
Supplement: Supplement 5 [file iovs-62-7-24_s005.pdf]

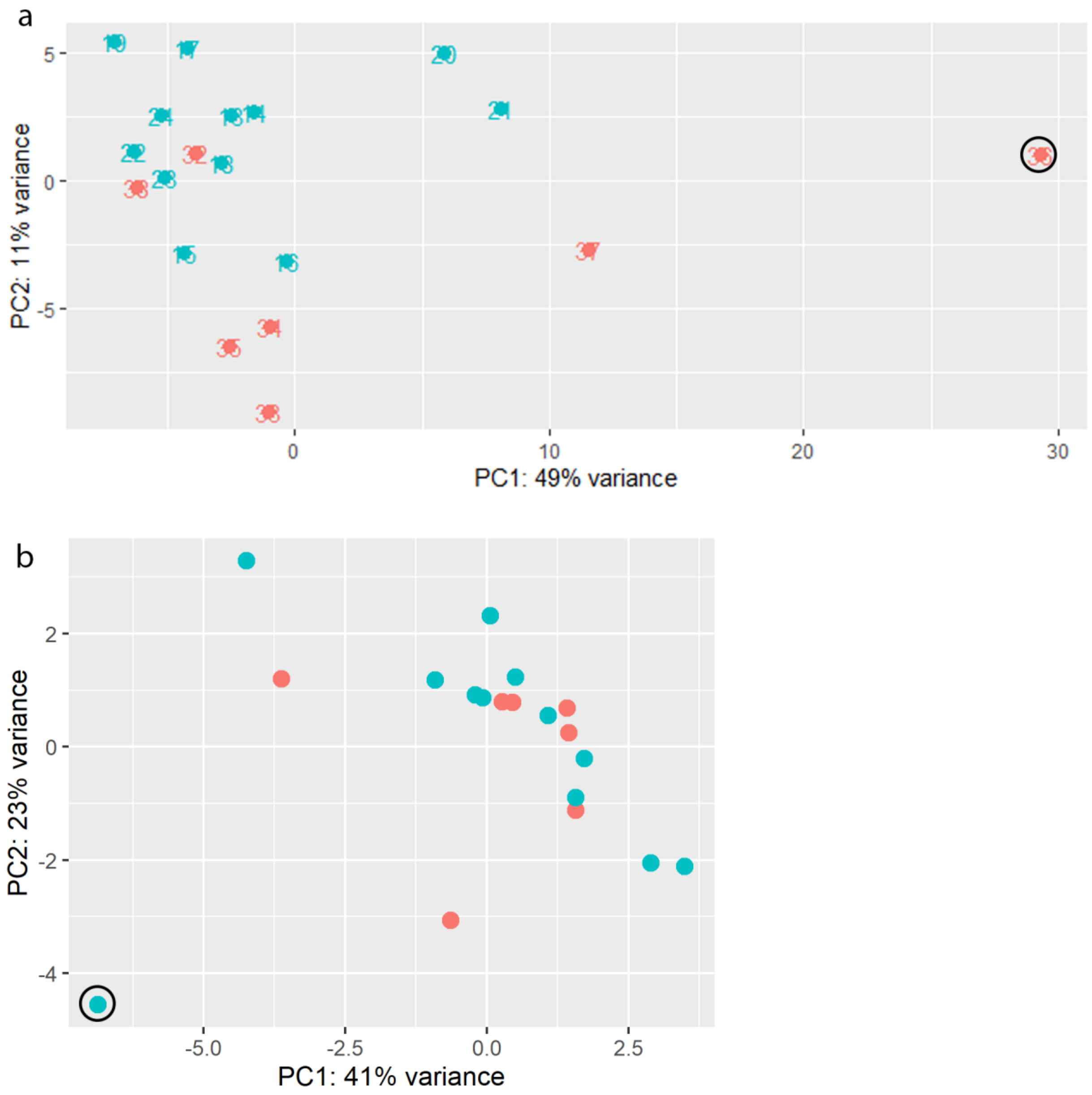

Supplemental figure 1: Principal component analysis of the AH samples (a) and of the serum samples (b). Library preparation was performed in two batches as indicated with the colors. In both AH and serum one sample, highlighted in black, was clearly an outlier and excluded from further analysis.

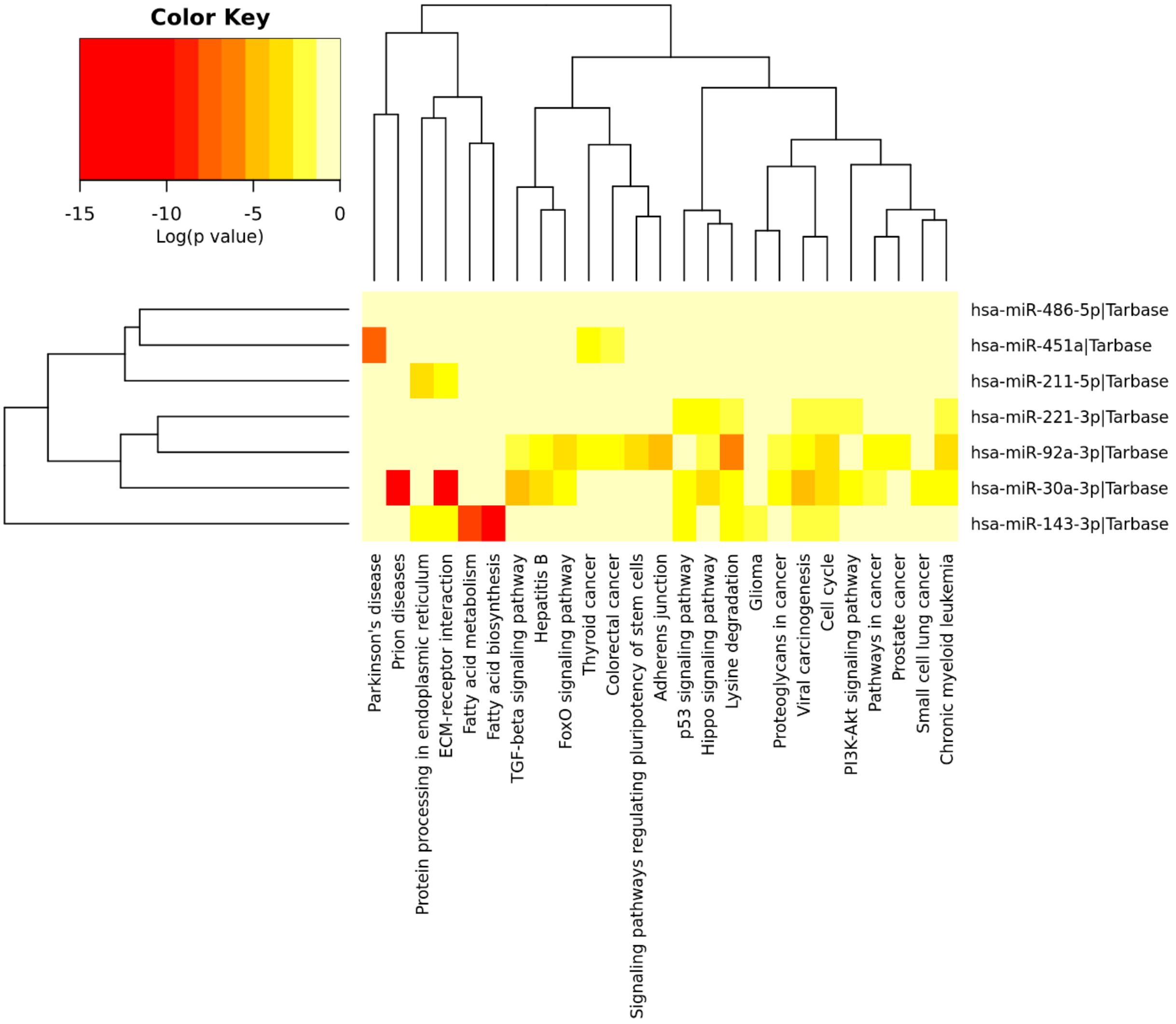

Supplemental figure 2. DIANA-miRPath (v3)* generated heatmap of pathways significantly enriched by gene targets of our reported miRNA.

*Vlachos IS, Zagganas K, Paraskevopoulou MD, Georgakilas G, Karagkouni D, Vergoulis T, et al. DIANA-miRPath v3.0: deciphering microRNA function with experimental support. Nucleic Acids Res. 2015;43(W1):W460-6

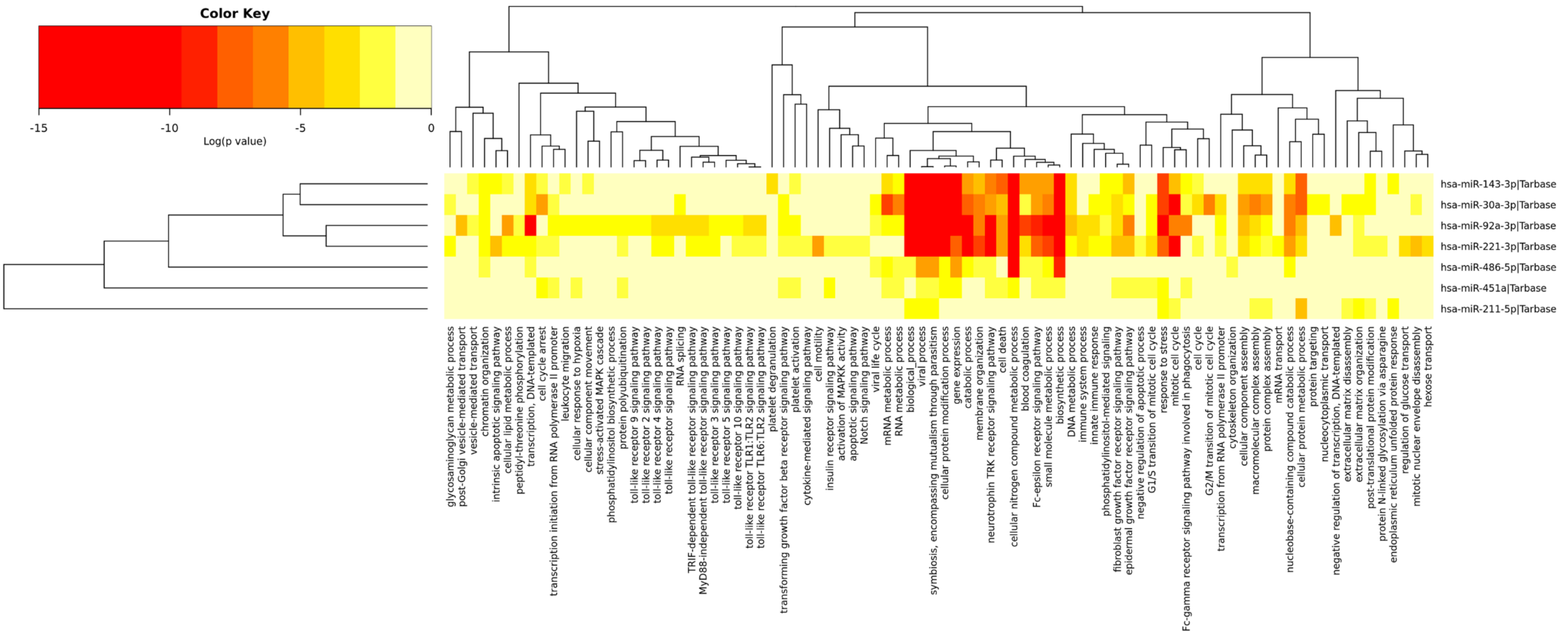

Supplemental figure 3: DIANA-miRPath (v3)* generated heatmap of biological processes (Gene Ontology) significantly enriched by gene targets of our reported miRNA.

*Vlachos IS, Zagganas K, Paraskevopoulou MD, Georgakilas G, Karagkouni D, Vergoulis T, et al. DIANA-miRPath v3.0: deciphering microRNA function with experimental support. Nucleic Acids Res. 2015;43(W1):W460-6
